# Aging-associated microstructural deterioration of vertebra in zebrafish

**DOI:** 10.1016/j.bonr.2019.100215

**Published:** 2019-07-17

**Authors:** Yasuyuki Monma, Yasuhito Shimada, Hiroko Nakayama, Liqing Zang, Norihiro Nishimura, Toshio Tanaka

**Affiliations:** a Department of Systems Pharmacology, Mie University Graduate School of Medicine, Tsu, Mie, Japan; b Department of Integrative Pharmacology, Mie University Graduate School of Medicine, Tsu, Mie, Japan; c Department of Bioinformatics, Mie University Advanced Science Research Promotion Center, Tsu, Mie, Japan; d Mie University Zebrafish Drug Screening Center, Tsu, Mie, Japan; e Graduate School of Regional Innovation Studies, Mie University, Tsu, Mie, Japan

**Keywords:** BMC, bone mineral content, BMD, bone mineral density, BV, bone volume, CT, Computed Tomography, FCV, first caudal vertebra, mpf, month-post-fertilisation, Tb, trabecular bone, Tb.N, trabecular number, Tb.Th, trabecular thickness, TV, tissue volume, V*m, tissue space star volume, V*tr, trabecular star volume, Micro CT, Aging, Teleost, Osteoporosis

## Abstract

Zebrafish, a small teleost fish, is currently emerging as an animal model of local and systemic aging. In this study, we assessed age-related degenerative changes in the vertebral bone of zebrafish (3–12 month-post-fertilisation [mpf]) using micro-CT scanning. The bone volume (BV) of the trabecular bone in the male and female fish peaked at 6 mpf and reduced with age. In contrast to BV, bone mineral density and tissue volume did not change after 6 mpf, implying that the total mineral volume in the trabecular area remains unchanged, retaining the strength of vertebra. In addition, we performed micro-structural analysis of the trabecular thickness, trabecular number, and star volume of the tissue space and trabeculae, and found that the size of the trabecular bone reduced with age. Furthermore, aged zebrafish (45 mpf) exhibited ectopic ossification inside or outside of their vertebrae.

In summary, we analysed bone structural parameters in adult zebrafish vertebra, which are also used in humans, and demonstrated that aged zebrafish have deteriorated microarchitecture (trabecular thickness and number, tissue space star volume and trabecular star volume) with reduction of trabecular bones, similar to that observed during aging in humans. Zebrafish can be utilised as an animal model to understand the pathology of human bone aging, and the discovery of new therapeutic agents against age-related osteoporosis.

## Highlights

• We analysed bone structural parameters in adult zebrafish vertebrae.
• Microstructural changes in aged-zebrafish are similar to those in humans.
• Aged zebrafish exhibited ectopic ossification inside or outside of their vertebrae.

## 1 Introduction

Bones exert mechanical and homeostatic functions in vertebrate animals, protecting the internal organs, enhancing locomotion and load-bearing, and serving as a reservoir of calcium homeostasis. With aging, these functions become impaired; bones become more fragile with the disturbance of mechanical functions and depletion of the calcium stores (Chan and Duque, 2002).

As a model animal, teleost fish have been used with growing success due to their many similarities with mammals in molecular pathways, and mechanisms involved in the onset of patterning and development of skeletal structures (Laizé et al., 2014). Anatomic and developmental features of teleost fish and mammalian skeletons are remarkably similar, with much of the skull, axial, and appendicular skeleton formed by identical bones, and highly conserved developmental events and underlying mechanisms of skeletogenesis, including the early formation of cartilage followed by bone formation (Javidan and Schilling, 2004). In contrast to worms and flies, zebrafish, a small vertebrate teleost possesses many structural and phenotypic similarities to humans, including the skeletal system (Schlegel and Stainier, 2007), and has been used to model a large number of human diseases because of the common patho-physiological pathways. The key regulators in bone formation have been highly conserved, and corresponding zebrafish orthologs share significant sequence similarities and overlapping expression patterns (Spoorendonk et al., 2010). Thus, there are several types of zebrafish models used for developmental diseases and discovery of osteogenic and osteotoxic drugs (Tarasco et al., 2017). While many studies have reported zebrafish models for developmental and/or congenital bone diseases along with for drug discovery targets, there are few reports concerning degenerative changes in the aging fish bone. Hayes AJ, et al. reported that micro-CT analysis in aged zebrafish (over 2-year-old) showed spinal deformity (or spinal curvature) similar to human osteoarthritis (Hayes et al., 2013). However, they did not conduct microstructural analyses of vertebra other than bone mineral density, which did not alter with aging.

In this study, to evaluate the use of zebrafish as a bone senescence model, we performed micro-CT analysis of vertebrae in adult zebrafish, and studied structural parameters in the trabecular and cortical bones, which correspond to mammalian parameters of bone morphological changes in degenerative disorders, such as osteoporosis. We tested 3–12-month-old male and female zebrafish and compared the data with aging models in humans and rodents.

## 2 Methods

### 2.1 Zebrafish

Zebrafish at 3, 6, 9, and 12 month-post-fertilisation (mpf) were allocated to four groups. Zebrafish (AB strain, the Zebrafish International Resource Center, Eugene, OR, USA) were kept at approximately 28 °C under a 14-h light and 10-h dark cycle. Water conditions of environmental quality were maintained according to the standard protocol. All animal procedures were approved by the Ethics Committee of Mie University, were performed according to the Japanese animal welfare regulation ‘Act on Welfare and Management of Animals’ (Ministry of Environment of Japan), and complied with international guidelines. For 3–12 mpf group, we used male and female zebrafish without apparent anomalies, e.g. spine curvature.

### 2.2 Micro-CT

Zebrafish were fixed in a stretched position on a sample holder. The 3-D micro-CT scan was performed with an in vivo System R_mCT 3D micro-CT scanner (Rigaku, Tokyo, Japan). The following settings were used: voltage, 90 kV; current, 150 μA; magnification, 20×; slice thickness (scanning width), 10 μm; and exposure time, 2 min according to our previous study (Hasumura et al., 2012). After scanning, image data were transferred to a workstation, and structural indices and degree of bone mineralisation were calculated using TRI/3D-BON software (Ratoc System Engineering, Tokyo, Japan). TRI/3D-BON builds 3-D models from serial tomographic datasets for visualisation and morphometric analysis of cancellous bone (Joo et al., 2003). The voxel size of the Micro-CT analysis is 10 μm. Images were reconstructed and viewed using i-View type R software (J. Morita Mfg., Kyoto, Japan).

### 2.3 Measurement of bone mineral density, bone mineral content, and bone volume/tissue volume

The first caudal vertebra was selected for analysis in the current study. Digital images were converted to 16-bit gray-scale TIFF format, using the Atlas TIFF Converter (Rigaku), and were imported into TRI/3D-Bon software. For bone mineral density (BMD) and bone mineral content (BMC) measurements, a hydroxyapatite calibration curve was prepared from images of phantoms (hydroxyapatite content; 200–1550 mg/cm^3^), and vertebrae were measured with the TRI/3D-Bon trabecular structure analysis routine using the obtained CT values (Nakada et al., 2014). Bone volume (BV) was calculated using tetrahedrons corresponding to the enclosed volume of the triangulated surface. Total tissue volume (TV) was the entire volume of the analysis. The normalised indices, trabecular bone volume fraction (BV/TV), were then calculated from these values.

### 2.4 Measurement of trabecular thickness and star volume

Trabecular thickness (Tb.Th) and trabecular number (Tb.N) were determined according to the previous studies (Hildebrand and Rüegsegger, 1997; Goulet et al., 1994). Tissue space star volume (V*m. space) [mm^3^] was calculated as previously reported (Vesterby et al., 1989; Vesterby, 1990). V*m. space was defined as the mean volume of a specific region of the tissue space in trabecular area that can be seen unobscured in all possible directions from a particular point within a tissue cavity. Trabecular star volume (V*tr) [mm^3^] was defined as the mean volume of a specific region that reaches in all possible directions from a particular point inside trabecular bone to its end.

### 2.5 Statistics

Statistical analyses were performed using Student's *t*-test, or one-way analysis of variance with the Bonferroni-Dunn multiple comparison procedure, depending on the number of comparisons, using GraphPad Prism version 8 (GraphPad Software Inc., San Diego, CA, USA). A *p*-value <0.05 denoted the presence of a statistically significant difference.

## 3 Results and discussion

### 3.1 Age-dependent decrease in BV, BMD, and BV/TV in the trabecular bone

**Fig. 1:**
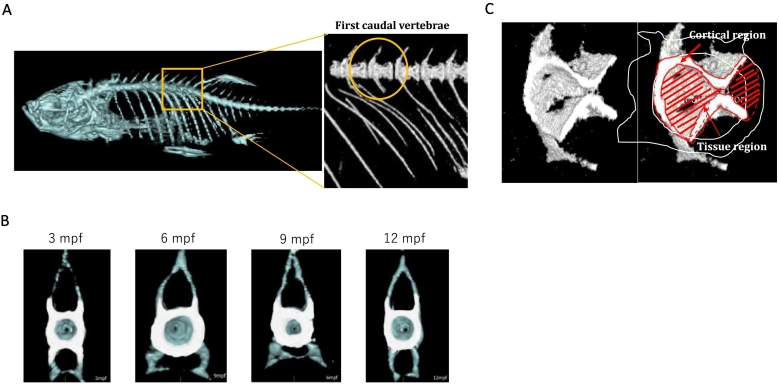
Morphological images with micro-CT visualisation. A. The position of FCV. FCV exhibits elongated unfused haemal arches with shortened ribs, and absence of a haemal spine (Bird and Mabee, 2003). We selected this vertebra in the following analyses. B. Cross-sectional images of FCV in 3–12 mpf zebrafish. Surrounding bone over the threshold of 300 mg/cm^3^ in BMD is defined as the cortical bone (white area), and the region surrounded by cortical bone is defined as ‘tissue’ (blue area), containing trabecular bone. C. Representative 3-D images of FCV.

For bone structural measurements, we focused on the first caudal vertebra (FCV) of zebrafish spine as previously reported (Bird and Mabee, 2003). FCV exhibits elongated unfused haemal arches with shortened ribs, and absence of a haemal spine (Fig. 1A). Fig. 1B and Fig. S1 show typical cross-sectional and sagittal images of 3–12 mpf female zebrafish. The body length of zebrafish was slightly increased by age (Fig. S2). After CT scan, FCV was divided into the cortical bone region (white area in Fig. 1B) and the portion surrounded by cortical bone (hereinafter “tissue”; blue area in Fig. 1B), according to the threshold of 300 mg/cm^3^ in BMD. Fig. 1C shows 3-D images of FCV, indicating cortical and tissue regions.

**Fig. 2:**
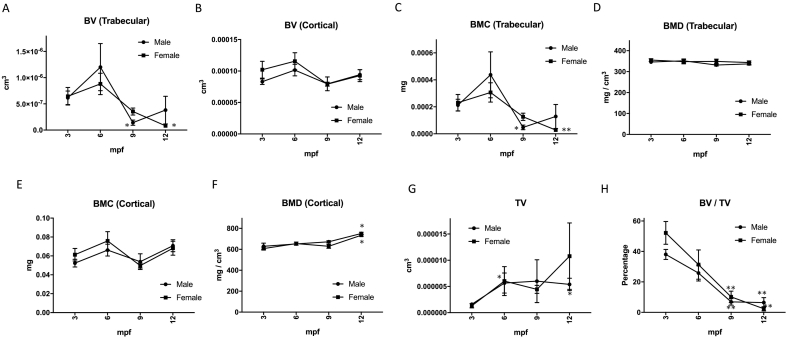
Morphometric parameters in aging zebrafish FCV. A–B. Trabecular (A) and Cortical (B) BV; C-D. BMC (C) and BMD (D) in trabecular bone; E–F. BMC (E) and BMD (F) in cortical bone; G–H. TV (G) and BV/TV (H). *p < 0.05, **p < 0.01 vs. 3 mpf male or female. *n* = 5–7. Error bars indicate ±SEM.

We first quantified BV in the trabecular and cortical bones from the CT images. In the trabecular bone, BV was drastically decreased in 9 mpf zebrafish in both sexes (Fig. 2A). However, such changes were not seen in the cortical BV with age (Fig. 2B). In human aging, the decrease of cortical BV in the vertebrae is less than that of trabecular BV (Chen et al., 2013), even in female menopause (Zebaze et al., 2010). In mammals, transition of BMC is parallel to BMD of the trabecular bone, and both decreased with aging in humans (Sievanen, 2000). Similar to humans, trabecular BMC decreased with aging in zebrafish (Fig. 2C). Because BMC is defined mainly by BV (Boyd et al., 1974), this result is quite understandable. However, in contrast to the trabecular BMC, the trabecular BMD was not altered with age (Fig. 2D). Additionally, the cortical BMC was not altered with age (Fig. 2E), while the cortical BMD increased (Fig. 2F). As the trabecular and cortical BMD in mammals decrease with age, these results seem a unique phenotype in zebrafish. In a previous study of aged zebrafish, BMD of the whole bone (the authors could not separate trabecular and cortical bone in vertebrae) was not altered with age, similar to our results (Hayes et al., 2013). No alterations in the trabecular BMD (Fig. 2D) with BV reduction (Fig. 2A) indicate that a reduced volume (or number) of trabecular bones (BV) in the vertebrae increases their relative mineral density (BMD), thus maintaining the strength of the whole spine. Furthermore, the increase in the cortical BMD (Fig. 2F) with unchanged BV (Fig. 2B) indicates that cortical bone size and strength increased with age, probably to compensate the reduction of trabecular bone strength.

As bone volume fraction (BV/TV) is a useful parameter for osteoporotic changes, we evaluated BV/TV in aged zebrafish. While tissue volume (TV; volume surrounded by cortical bone) showed a tendency (no significance) to increase with age (Fig. 2G), BV (trabecular)/TV was continuously decreasing with age in both males and females (Fig. 2H), quite similar to human aging and osteoporotic change (Legrand et al., 2000). The reduction of BV/TV indicates that the trabecular bone mass reduced drastically in the trabecular bone region.

### 3.2 3-D architectural analysis of osteoporotic changes in aged zebrafish

**Fig. 3:**
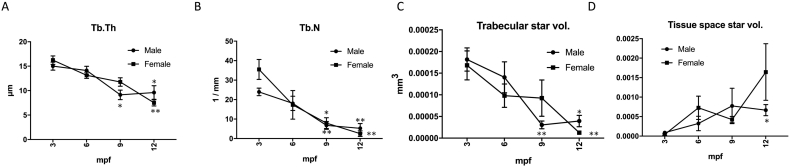
3-D architectural parameters in aging zebrafish FCV. A. Structural thickness of trabecular bone (Tb.Th); B. Numbers of trabecular bone (Tb.N); C. Trabecular star volume; and D. Tissue space star volume. *p < 0.05, **p < 0.01 vs. 3 mpf male or female. *n* = 5–7. Error bars indicate ±SEM.

To architecturally analyse zebrafish bone, we quantified structural Tb.Th and Tb.N, according to a human CT study (Goulet et al., 1994). It was observed that both Tb.Th and Tb.N decreased with age (Fig. 3A and B), indicating that the trabecular bone became thinner and fewer with aging in zebrafish vertebra, similar to humans (Cui et al., 2008). In addition, the Tb.Th and Tb.N values in zebrafish were quite different from those in humans. For example, Tb.Th in healthy human vertebrae (around 65-year-old) (Mullender et al., 2005), was about 135 μm (male, 133 μm; female, 138 μm), 10-times more than that of 12 mpf zebrafish (male, 9.6 μm; female, 7.5 μm). Similarly, human Tb.N was about 1.5 mm^−^^1^ (male, 1.47 mm^−^^1^; female, 1.66 mm^−^^1^) (Vesterby et al., 1989), lesser than that of 12 mpf zebrafish (male, 5.3 mm^−^^1^; female, 2.5 mm^−^^1^). Even after considering the difference in body size between zebrafish and humans (>50-times in the body length), zebrafish have a thicker trabecular bone in the vertebral column, compared to humans. It is believed that aquatic animals are usually less affected by the gravity, because of higher density of water compared to air, and hence have ‘weak’ bone structure. However, our results indicate that zebrafish have a more robust trabecular microstructure than humans, which may allow them to swim rapidly in conditions where water resistance is higher than standard gravity.

The age-dependent decrease in the amount of the trabecular bone can not only be explained by thinning of the trabecular bone, but also must be due to a loss of structural integrity, leading to a discontinuity in the trabecular network. The star volume analysis (V*tr and V*m. space) provides an absolute and reasonably precise estimate of the size of the trabecular and marrow space in three-dimensional terms (Vesterby et al., 1989). Because zebrafish do not have bone marrow in the vertebrae, we defined V*m. space as tissue space star volume. In our results, V*tr was reduced with age (Fig. 3C), indicating that the size of trabecular bones became smaller and thinner, similar to Tb.Th (Fig. 3A), and with the results of first lumbar vertebra in aged human study (Thomsen et al., 2002). In addition, the 9 mpf female showed a larger V*tr than the male at the same age (9.2 × 10^−^^5^ mm^3^ in female vs. 3.1 × 10^−^^5^ mm^3^ in male, *p* < 0.1), however at 12 mpf, the V*tr of female and male were almost equal. This implies the involvement of female hormones to keep V*tr in early stage of aging (6–9 mpf). Contrary to V*tr values, V*m. space increased with age (Fig. 3D), indicating that unobscured (no trabecular bone) space in the tissue cavity was enlarged. Larger V*m. space indicates lesser trabecular bone connectivity due to aging, as zebrafish lost trabecular thickness (Fig. 3A), number (Fig. 3B), and volume (Fig. 3C). Interestingly, 12 mpf males had larger tissue space compared to females of the same age (1.6 × 10^−^^3^ mm^3^ in female vs. 0.6 × 10^−^^3^ mm^3^ in male, *p* < 0.1), which might be caused by the reduction in female hormones.

### 3.3 Microstructural phenotypes in super-aged zebrafish

**Fig. 4:**
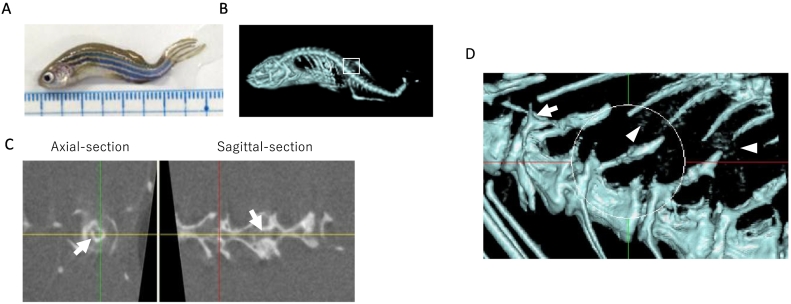
Micro-CT images of 45 mpf zebrafish. A–B. Whole bright field (A) and micro-CT (B) images of aged zebrafish; C. Axial and sagittal-sectional image of FCV, Arrows indicate ossification inside FCV; D. Magnified image of B (white circle is FCV), Arrowheads indicate ectopic ossifications around vertebrae.

Gerhard GS, et al. reported that the mean life span of zebrafish (outbred) was about 42 months, with a sharp decline in survival at about 40 mpf (Gerhard et al., 2002). In our laboratory, the life span of zebrafish was shorter, with a mean life span about 35 mpf. Almost all fish over 30 mpf showed a common age-related spinal curvature (Fig. 4A), as reported previously (Hayes et al., 2013). Fig. 4B shows micro-CT images of 45 mpf female zebrafish. Only 3 females survived to this age, and they all showed spinal curvature, as previously described (Gerhard et al., 2002). By CT analysis, we found that these 3 fish had intra-vertebrae ossification in the trabecular bone region (Fig. 4C, white arrows). We also performed structural analysis of trabecular bone in these 45 mpf zebrafish as described above; however, the intra-vertebrae ossification hindered the accuracy of the results. In addition, these super-aged zebrafish showed ectopic calcification (Fig. 4D, arrowheads) around regular bones. In humans, chronic kidney disease is one of the common causes of ectopic calcification in elderly people (Moe and Chen, 2008). Similarly, high dietary phosphate-induced ectopic calcification and bone abnormalities have been reported in mice with chronic kidney disease (Lau et al., 2013). Thus, we hypothesised that kidney senescence is one of the important factors in determining microstructural trabecular bone phenotypes, but needs to be elucidated further. In the cortical bone, BMD increased in 45 mpf (796.3 ± 89.7 mg/cm^3^) compared to 12 mpf (734.3 ± 20.0 mg/cm^3^) female zebrafish. This suggests that mineral density of the cortical bone region increases throughout the life to strengthen its structure, instead of faltering with age, as we hypothesised from Fig. 2F.

## 4 Summary

Zebrafish have been proven as an excellent model organism for the musculoskeletal system in developmental biology, congenital human diseases, and aging. However, due to the small size of fish vertebrae, there are no reports studying the zebrafish bone microstructure, as elaborated in mammals. To the best of our knowledge, this is the first study to quantify the structural parameters, and demonstrate the use of zebrafish as a model organism for bone senescence (Table 1), especially for 3-D architectural studies. In fact, the limitation of the voxel size of our micro-CT machine (10 μm) omits the bone with thickness <20 μm, theoretically. Further improvement of micro-CT resolution is needed. With the development of oral administration protocol for test compounds (Zang et al., 2011), zebrafish may serve as a model organism for long-term chemical screening against senescence-associated bone deterioration, including menopausal osteoporosis, along with immunohistochemistry or gene expression analysis.

**Table 1:** Comparison of bone parameters between zebrafish and humans in aging.

		Zebrafish	Human
BV	Trabecular	Decreased	Decreased
	Cortical	No change	Slightly decreased
BMC	Trabecular	Decreased	Decreased
	Cortical	No change	Decreased
BMD	Trabecular	No change	Decreased
	Cortical	Increased	Decreased
3-D architecture	TV	Increased	Increased
	BV/TV	Decreased	Decreased
	Tb.Th	Decreased	Decreased
	Tb.N	Decreased	Decreased
	V*tr	Decreased	Decreased
	V*m	Increased	Increased

## Transparency document

Transparency document Image 1

## Declaration of Competing Interest

The authors declare no conflict of interest.

## Appendix A Supplementary data

Supplementary figures Image 2
