# Supplementary material for: Aging-associated microstructural deterioration of vertebra in zebrafish
Source: Bone Rep. 2019 Jul 17;11:100215. doi: 10.1016/j.bonr.2019.100215 (PMC6676153; doi:10.1016/j.bonr.2019.100215)
Supplement: Supplementary file 2 — Supplementary figures [file mmc2.docx]

**Supplementary Figures**

**Figure S1**


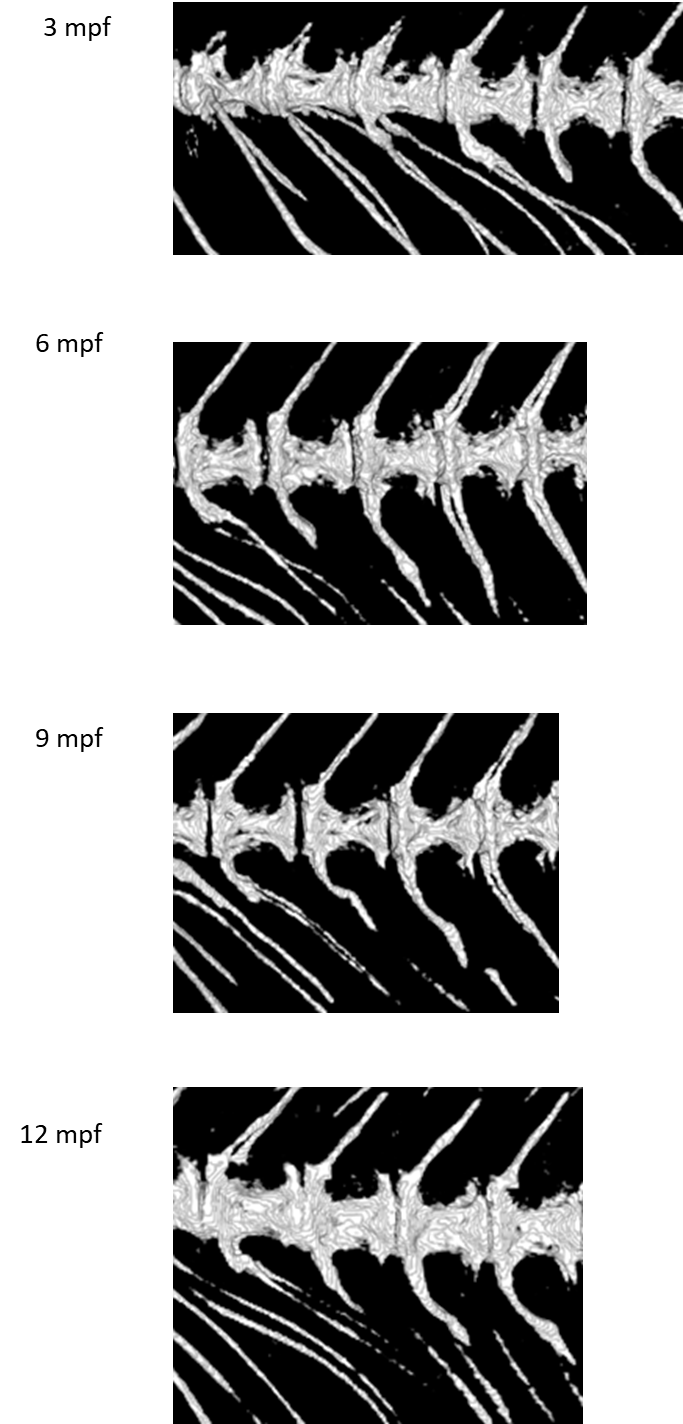


**Figure S1. Sagittal images of vertebrae in 3 – 12 mpf female zebrafish.**

**Figure S2**

**
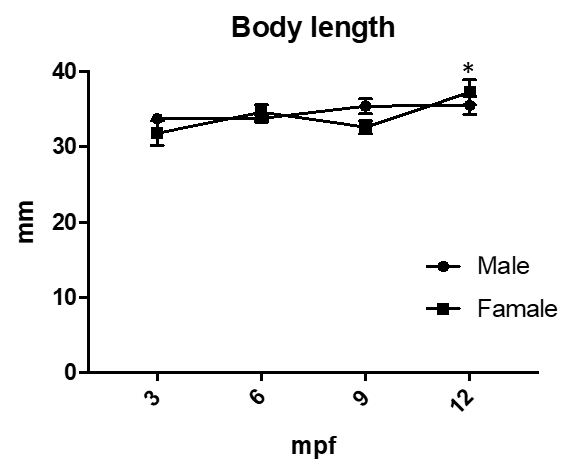
**

**Figure S2. Body length of 3 – 12 mpf zebrafish.** **p* < 0.05 vs. 3 mpf male or female. n = 5-7. Error bar indicates ±SEM.
